# *CENPE* expression is associated with its DNA methylation status in esophageal adenocarcinoma and independently predicts unfavorable overall survival

**DOI:** 10.1371/journal.pone.0207341

**Published:** 2019-02-04

**Authors:** Xueqiang Zhu, Xing Luo, Gang Feng, Hui Huang, Yangke He, Wen Ma, Changqing Zhang, Ming Zeng, Hao Liu

**Affiliations:** 1 Cancer Center, Sichuan Academy of Medical Sciences & Sichuan Provincial People’s Hospital, School of Medicine, University of Electronic Science and Technology of China, Chengdu, China; 2 Division of Thoracic Surgery, Department of Surgery, Sichuan Academy of Medical Sciences & Sichuan Provincial People’s Hospital, School of Medicine, University of Electronic Science and Technology of China, Chengdu, China; 3 Department of Tumor Center, Gansu Provincial People's Hospital, Lanzhou, China; Sapporo Ika Daigaku, JAPAN

## Abstract

Centrosome-associated protein E (CENPE) is a plus end-directed kinetochore motor protein, which plays a critical role in mitosis. In this in silico study, using data from the Cancer Genome Atlas-Esophageal Carcinoma (TCGA-ESCA), we analyzed the expression profile of *CENPE* mRNA in esophageal squamous cell carcinoma (ESCC) and adenocarcinoma (EA), its independent prognostic value and the potential mechanisms of its dysregulation in EA. Results showed that both ESCC and EA tissues had significantly elevated *CENPE* expression compared with their respective adjacent normal tissues. However, Kaplan-Meier survival curves showed that high *CENPE* was associated with unfavorable OS in EA. Univariate and multivariate analysis confirmed that *CENPE* expression was an independent indicator of unfavorable OS in EA patients, as a continuous variable (HR: 1.861, 95%CI: 1.235–2.806, *p* = 0.003) or as categorical variables (HR: 2.550, 95%CI: 1.294–5.025, *p* = 0.007). However, *CENPE* expression had no prognostic value in ESCC. Compared with the methylation status in normal samples, 3 CpG sites were hypomethylated (cg27388036, cg27443373, and cg24651824) in EA, among which two sites (cg27443373 and cg24651824) showed moderately negative correlation with *CENPE* expression. In addition, we also found that although heterozygous loss (-1) was frequent in EA (50/88, 56.8%), it was not necessarily associated with decreased *CENPE* expression compared with the copy neutral (0) cases. The methylation of the -1 group was significantly lower than that of the +1/0 group (*p* = 0.04). Based on these findings, we infer that *CENPE* upregulation in EA might serve as a valuable indicator of unfavorable OS. The methylation status of cg27443373 and cg24651824 might play a critical role in modulating *CENPE* expression.

## Introduction

Esophageal squamous cell carcinoma (ESCC) and adenocarcinoma (EA) are the two major histologic types of malignant esophageal neoplasms [1]. Although ESCC accounts for most (about 90%) of the esophageal neoplasms, the incidence rate of EA has been rising in some western countries due to the growing prevalence of some EA associated risk factors, such as gastroesophageal reflux, smoking and obesity [CENPE]. Since early esophageal cancer may be totally asymptomatic, most of the patients were diagnosed with advanced tumors. The overall 5-year survival rate is lower than 20% in both ESCC and EA in the United States [1].

These two subtypes have distinct origins and molecular mechanisms [2, 3]. ESCC begins in flat cells lining the esophagus, while EA usually occurs just above the esophagogastric junction and begins in the cells of mucus-secreting glands. One recent study showed that ESCC showed stronger molecular similarities to SCCs in other organs, while EA presented a strong molecular resemblance to chromosomally unstable gastric adenocarcinoma [3]. Therefore, although the ESCC and EA have similar 5-year survival rate, it is necessary to explore the specific prognostic indicators in different subtypes of esophageal cancer.

Centrosome-associated protein E (CENPE) is a plus end-directed kinetochore motor protein, which belongs to the kinesin-7 subfamily and plays a critical role in mitosis [4]. CENPE accumulates in the G2 phase of the cell cycle and plays an essential role in transporting pole-proximal chromosomes to the spindle equator during prometaphase [5], the formation of stable kinetochore-microtubule attachment during metaphase [6], and the microtubule plus-end elongation [7]. Knockdown of *CENPE* results in increased frequency of chromosome misalignment, lagging chromosomes and subsequent delayed mitotic progression in normal cells [8, 9].

In human tissues, *CENPE* mRNA expression shows a strong association with cell proliferation [10]. *CENPE* was aberrantly upregulated in multiple types of cancer and was associated with facilitated cell-cycle progression and tumor cell growth, such as in epithelial ovarian cancer [11], prostate cancer [12] and triple-negative breast cancer [13]. However, in esophageal cancer, the expression profile of *CENPE* mRNA and its prognostic value have not been explored.

In this study, using data from the Cancer Genome Atlas-Esophageal Carcinoma (TCGA-ESCA), we analyzed the expression profile of *CENPE* mRNA in ESCC and EA, its independent prognostic value in terms of overall survival (OS) and the potential mechanisms of its dysregulation in EA.

## Materials and methods

This study was an in silico retrospective analysis based on data from publicly available databases. Thus no ethical approval and patient consent are required.

### Secondary analysis using data from TCGA-ESCA

The clinicopathological, genetic and survival data in TCGA-ESCA were obtained by using the UCSC Xena browser (https://xenabrowser.net/). In this dataset, 96 cases of ESCC (with 3 cases of adjacent normal tissues) and 89 cases of EA (with 15 cases of adjacent normal tissues) were included. The information of the patients, such as their age, gender, race, ethnicity, and vital status was available in https://portal.gdc.cancer.gov/projects/TCGA-ESCA. None of the patients received neoadjuvant treatment. The flowchart showing data availability among the patients was given in S1 Fig.

The clinicopathological, survival and genetic data, including age at diagnosis, gender, histologic grade, smoking history, reflux history, Barrett’s esophagus, pathologic stage, history of esophageal cancer, radiation therapy, postoperative drug therapy, residual tumor after primary therapy, primary therapy outcome, recurrence status, living status, OS in days, RNA-seq data of *CENPE* expression, *CENPE* DNA copy number alterations (CNAs) (calculated by gene-level thresholded Genomic Identification of Significant Targets in Cancer 2.0 (GISTIC2)) and *CENPE* DNA methylation (Methylation450k) (measured by Infinium HumanMethylation450 BeadChip) were downloaded. CNAs were defined as homozygous deletion (-2), heterozygous loss (-1), copy-neutral (0), low-level copy gain (+1), high-level amplification (+2).

### Immunohistochemistry (IHC) staining of CENPE

Human paraffin-embedded EA/adjacent normal tissue array was purchased from Alenabio (ES781, Xian, China), which includes 13 cases of EA and 10 cases of adjacent normal tissues. Briefly, the tissue array was treated with 3% H_2_O_2_ for 10 min to inactivate tissue peroxidases were inactivated. Then, the array was pre-treated with antibody diluent solution containing 1% BSA, followed by 20 min incubation at room temperature with primary antibodies against CENPE (1:100 dilution, 28142-1-AP, Proteintech Group, Wuhan, China). Labeling was accomplished with biotinylated secondary antibodies (SP-9001, ZSGB-BIO, Beijing, China) and DAB kit (ZSGB-BIO), and counterstained with hematoxylin for 5 min. Protein staining score was given by two experienced pathologists, who do not have authorship in this study. The scoring system follows the method recommended by the Human Protein Atlas (HPA), with regard to staining intensity (negative, weak, moderate or strong) and fraction of stained cells (<25%, 25–75% or >75%) [14, 15]. The combination of intensity and fractions is automatically converted into protein expression level scores, which including not detected, low, medium and high.

### In silico analysis using cBioPortal for cancer genomics and string

The genes strongly co-expressed with *CENPE* in EA (Pearson’s r≥0.6 and Spearman’s r≥0.6) were identified using cBioPortal for Cancer Genomics [16]. Then, the potential molecular interactions between these genes were identified using String 10.5 (https://string-db.org/). By setting 0.4 as the minimum required interaction score.

### Statistical analysis

Statistical analysis was performed by using GraphPad Prism 6.0 (GraphPad Inc., La Jolla, CA, USA) or SPSS 19.0 software package (SPSS Inc., Chicago, IL, USA). Welch’s unequal variances t-test was performed to examine the difference in *CENPE* expression or DNA methylation. The association between *CENPE* expression and the clinicopathological parameters in EA patients was assessed by using the Chi-squared test by two-sided Fisher’s exact test. Kaplan-Meier curves of OS and RFS were generated using GraphPad Prism 6.0. The best cutoff (Youden Index) of *CENPE* expression/*CENPE* DNA methylation in receiver operating characteristic curve (ROC) for death and recurrence detection were identified and used as the cutoff in Kaplan-Meier survival curves. Log-rank test was conducted to examine the significance of the difference between the curves. Univariate and multivariate Cox regression models were used to evaluate the prognostic significance of *CENPE* expression, as category variables or as a continuous variable. Linear regression analysis was performed to assess the correlation between *CENPE* expression and the methylation of the CpG sites in its DNA. *p*<0.05 was considered statistically significant.

## Results

### *CENPE* was significantly upregulated in both ESCC and EA tissues compared with their respectively adjacent normal tissues

**Fig 1:**
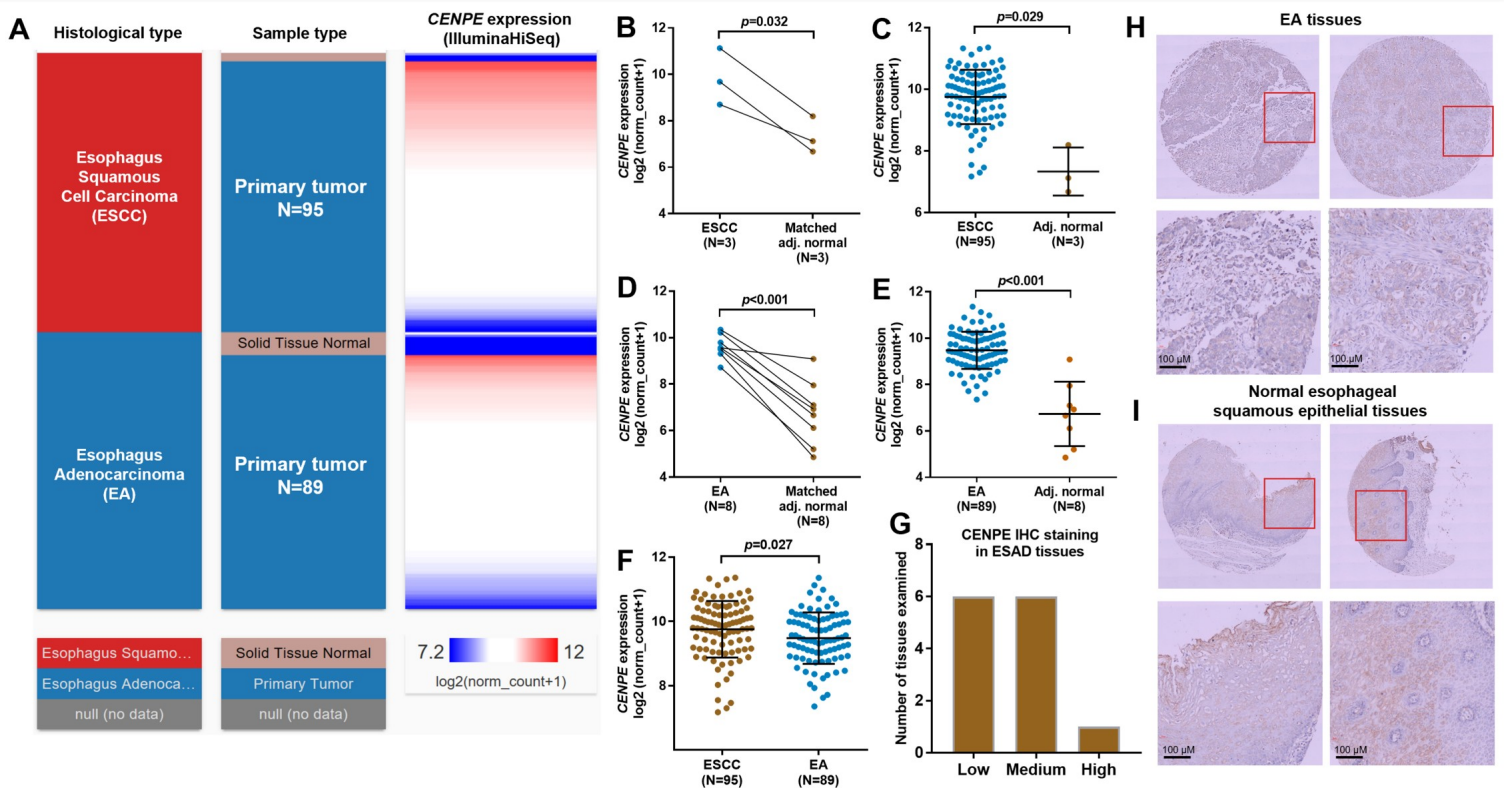
*CENPE* was significantly upregulated in both ESCC and EA tissues compared with their respective adjacent normal tissues. **A.** A heatmap showing the expression of *CENPE* in both ESCC and EA tissues and their respective adjacent normal tissues. **B-F.** Comparison of *CENPE* expression between ESCC/EA and the matched adjacent normal tissues (B and D), between all ESCC/EA tissues and the adjacent normal tissues (C and E) and between ESCC and EA tissues (F). **G.** CENPE IHC staining score summary in the 13 cases of EA tissues examined. **H-I.** Representative images of CENPE staining in EA (H) and normal esophageal squamous epithelial tissues (I).

Using RNA-seq data in TCGA-ESCA, we examined the expression profile of *CENPE* RNA in ESCC and EA tissues compared with their respectively adjacent normal tissues (Fig 1A). Statistical analysis showed that both ESCC and EA tissues had significantly elevated *CENPE* expression compared with matched adjacent normal tissues (*p* = 0.032 and *p*<0.001 respectively) (Fig 1B and 1D). These trends were confirmed between all available cancer tissues and the adjacent normal tissues (*p* = 0.029 and *p*<0.001 respectively) (Fig 1C and 1E). Besides, *CENPE* expression was higher in ESCC tissues than in EA tissues (Fig 1F). Then, using commercially available EA tissue array, we examined the expression of CENPE in 13 cases of EA tissues by IHC staining. Results showed that all the EA tissues had positive CENPE expression, which include 6 low, 6 medium and 1 high expression (Fig 1G). Representative images showed that CENPE had both nuclear and cytoplasm distribution in EA cells (Fig 1H). Although EA does not arise from esophageal squamous epithelial cells, available data in the HPA showed that they have positive CENPE expression [14, 15]. In this study, we confirmed CENPE expression in the normal cells, which help to serve as a positive control of the primary antibody used (Fig 1I).

### High *CENPE* was associated with unfavorable OS in EA, but not in ESCC patients

**Fig 2:**
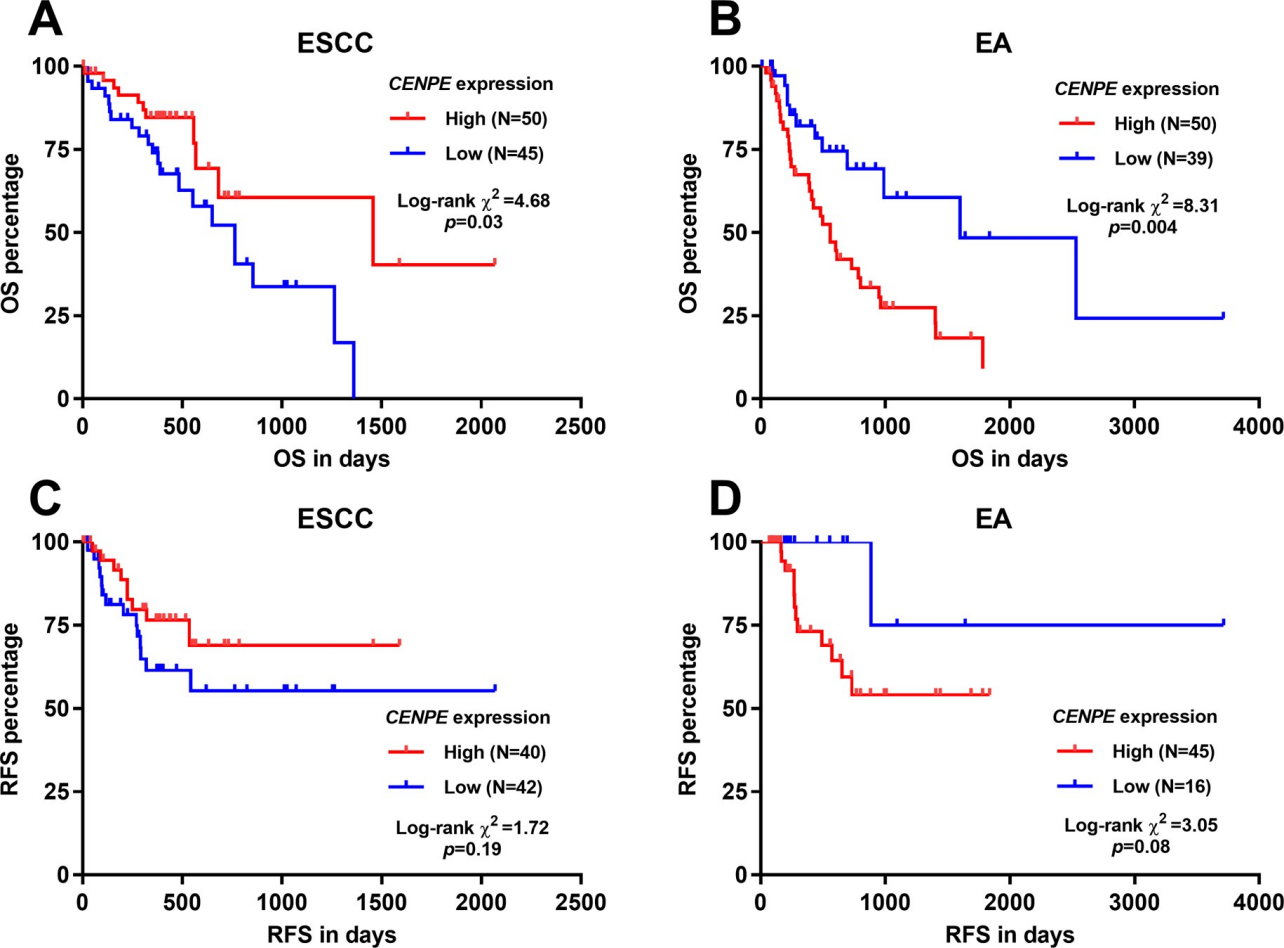
Kaplan-Meier curves of OS/RFS in ESCC (A) and EA (B) patients. ESCC (A and C) and EA (B and D) patients were divided into two groups by the best cutoff of *CENPE* expression.

By generating Kaplan-Meier curves of OS, we assessed the association between *CENPE* expression and OS in ESCC and EA respectively. Results showed that under the best cutoff model, *CENPE* expression was associated with better OS in ESCC patients (Fig 2A, *p* = 0.03), but was associated with significantly shorter OS in EA patients (*p* = 0.004, Fig 2B). However, *CENPE* expression was not associated with RFS in either ESCC (Fig 2C, *p* = 0.19) or EA (*p* = 0.08) (Fig 2D).

### *CENPE* expression was an independent prognostic indicator in terms of OS in EA

Then, we analyzed the association between *CENPE* expression and the clinicopathological parameters in EA patients (Table 1). Results showed that the high *CENPE* expression group was associated with a higher ratio of death compared to the low *CENPE* expression group (28/44 vs. 17/45, *p* = 0.02) (Table 1). However, no other associations were observed. In univariate analysis, we found that high histologic grade (G3), advanced pathologic stage and *CENPE* expression (as either a continuous variable or categorical variables) were associated with unfavorable OS in EA patients (Table 2). However, *CENPE* expression was not a risk factor of OS in ESCC patients in univariate analysis (*p* = 0.12) (S1 Table). Multivariate analysis confirmed that *CENPE* expression was an independent indicator of unfavorable OS in EA patients, no matter as a continuous variable (HR: 1.861, 95%CI: 1.235–2.806, *p* = 0.003) or as categorical variables (HR: 2.550, 95%CI: 1.294–5.025, *p* = 0.007) (Table 2). However, *CENPE* expression had no prognostic value in terms of RFS in either EA or ESCC (*p* = 0.611 and 0.765 respectively) (S2 Table).

**Table 1:** The association between *CENPE* expression and clinicopathological parameters in EA patients in TCGA-ESCA.

Parameters		*CENPE* expression		*p-*value
		High (N = 44)	Low (N = 45)	
**Age (Mean ± SD)**		66.18±1.73	67.51±1.89	0.61
**Gender**	Female	6	6	1.00
	Male	38	39	
**Histologic grade**	G1/G2	14	17	0.30
	G3	17	11	
	No data	13	17	
**Barrett’s esophagus**	No	26	28	0.82
	Yes	15	13	
	No data	3	4	
**Smoking history**	2/3/4	23	27	0.81
	1	12	12	
	no data	9	6	
**Reflux history**	No	14	19	0.35
	Yes	24	19	
	No data	6	7	
**Pathologic stage**	III/IV	15	19	0.34
	I/II	20	15	
	Discrepancy/no data	9	11	
**History of esophageal cancer**	No	24	25	0.71
	Yes	5	3	
	No data	15	17	
**Radiation therapy**	No	32	36	0.73
	Yes	5	4	
	No data	7	5	
**Postoperative drug therapy**	No	29	37	0.11
	Yes	8	3	
	No data	7	5	
**Residual tumor**	R0	29	30	1.00
	R1	4	4	
	RX/no data	11	11	
**Primary therapy outcome**	PD+SD	3	6	0.44
	CR+PR	14	12	
	no data	27	27	
**Recurrence status**	No	23	25	0.76
	Yes	7	6	
	no data	14	14	
**Living Status**	Living	16	28	0.02
	Dead	28	17	

G1: well differentiated (low grade); G2: moderately differentiated (intermediate grade); G3: poorly differentiated (high grade). Smoking history: 1: lifelong non-smoker; 2: current smoker; 3. Current reformed smoker (for>15 yrs); 4. Current reformed smoker (for≤15 yrs). R0: No residual tumor; R1: Microscopic residual tumor; RX: The presence of residual tumor cannot be assessed. CR: complete response; PR: partial response; SD: stable disease; PD: progressive disease.

**Table 2:** Univariate and multivariate analysis of OS in EA patients in TCGA-ESCA.

Parameters	Univariate analysis				Multivariate analysis			
	*p*	HR	95%CI (lower/upper)		*p*	HR	95%CI (lower/upper)	
***CENPE* as a continuous variable**								
**Age**	0.269	0.987	0.965	1.010				
**Gender** Female vs Male	0.963	0.976	0.345	2.755				
**Histologic grade** G3 *vs*. G1/G2	**0.018**	2.357	1.157	4.804	0.068	2.020	0.950	4.296
**History of esophageal cancer** No *vs*. Yes	0.907	1.062	0.392	2.875				
**Barrett’s esophagus** No *vs*. Yes	0.873	0.949	0.503	1.794				
**Pathologic stages** III/IV *vs*. I/II	**0.002**	3.353	1.579	7.119	**0.001**	3.854	1.753	8.475
**Residual tumor** No *vs*. Yes	0.069	0.451	0.192	1.063				
**Reflux History** No *vs*. Yes	0.759	1.114	0.560	2.215				
**Smoking History** Yes *vs*. No	0.414	1.376	0.640	2.957				
**Postoperative drug therapy** No *vs*. Yes	0.891	0.940	0.392	2.257				
**Radiation therapy** No *vs*. Yes	0.581	1.339	0.475	3.776				
**Primary therapy outcome success** SD+PD *vs*. CR+PR	0.645	0.737	0.202	2.696				
***CENPE* expression**	**0.011**	1.639	1.118	2.403	**0.003**	1.861	1.235	2.806
***CENPE* as categorical variables**								
**Histologic grade** G3 *vs*. G1/G2	**0.018**	2.357	1.157	4.804	0.143	1.790	0.821	3.903
**Pathologic stages** III/IV *vs*. I/II	**0.002**	3.353	1.579	7.119	**<0.001**	4.178	1.902	9.179
***CENPE* expression** High *vs*. Low	**0.027**	2.007	1.084	3.713	**0.007**	2.550	1.294	5.025

### *CENPE* expression was negatively correlated with its DNA methylation in EA

**Fig 3:**
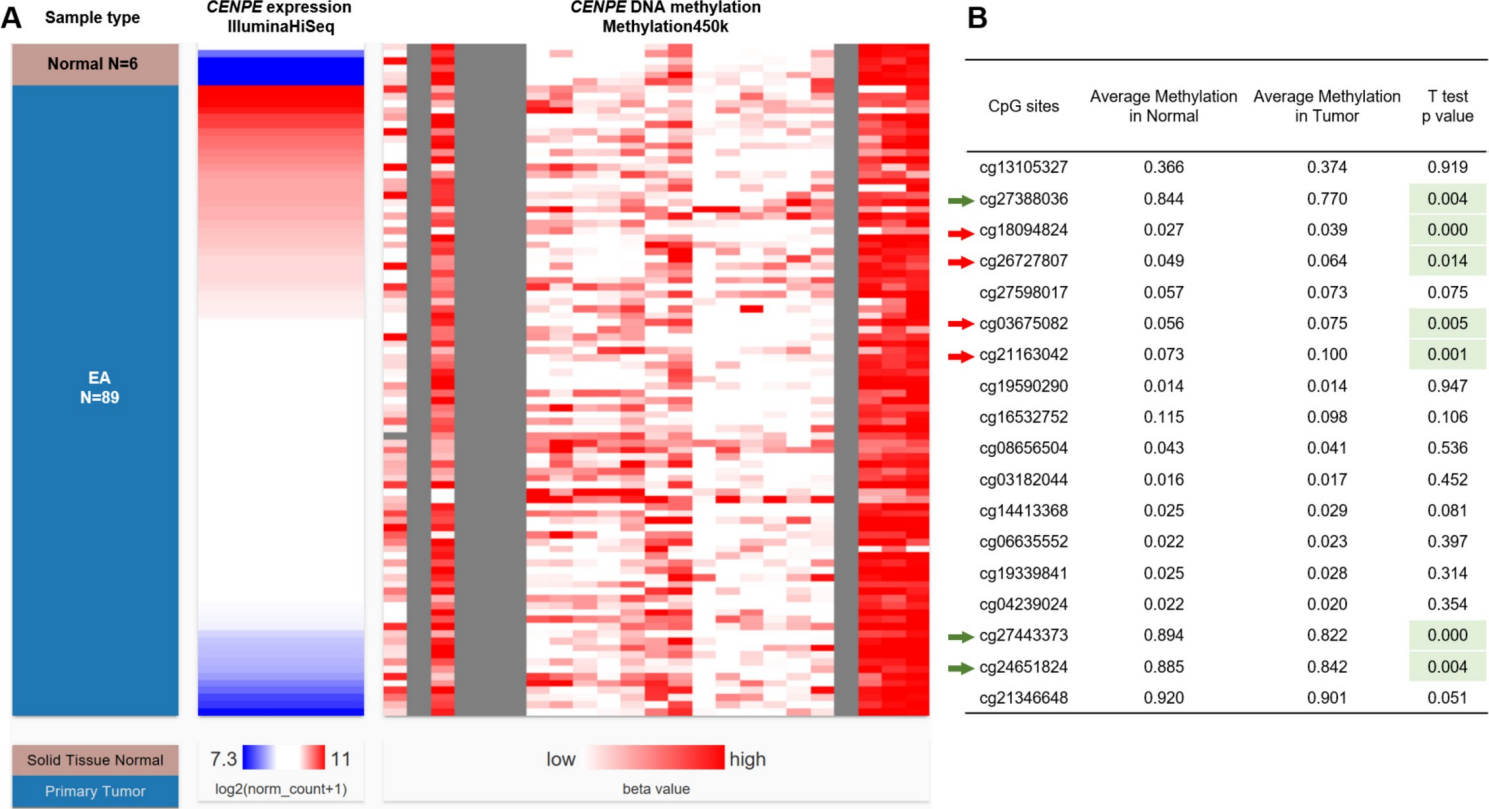
Comparison of *CENPE* methylation between EA and the adjacent normal tissues. **A-B.** A heatmap (B) and a statistical comparison summary (B) showing the correlation between *CENPE* expression and the methylation status of the 18 CpG sites in *CENPE* DNA in EA (N = 89) and adjacent normal tissues (N = 6).

*CENPE* DNA methylation status of 6 normal samples and 98 EA samples was examined by Infinium HumanMethylation450 BeadChip, which covered 18 CpG sites in *CENPE* DNA (Fig 3A). Compared with the methylation status in the 6 normal samples, 4 sites (cg18094824, cg26727807, cg03675082 and cg21163042) were hypermethylated (red arrows, Fig 3B), while 3 sites were hypomethylated (cg27388036, cg27443373, and cg24651824) in EA (green arrows, Fig 3B). Noticeably, the overall methylation status of the 4 hypermethylated sites was still relatively low (methylation value ≤0.1) in EA samples (Fig 3B). In comparison, the 3 hypomethylated sites were highly methylated in normal tissues (methylation value >0.84) (Fig 3B).

**Fig 4:**
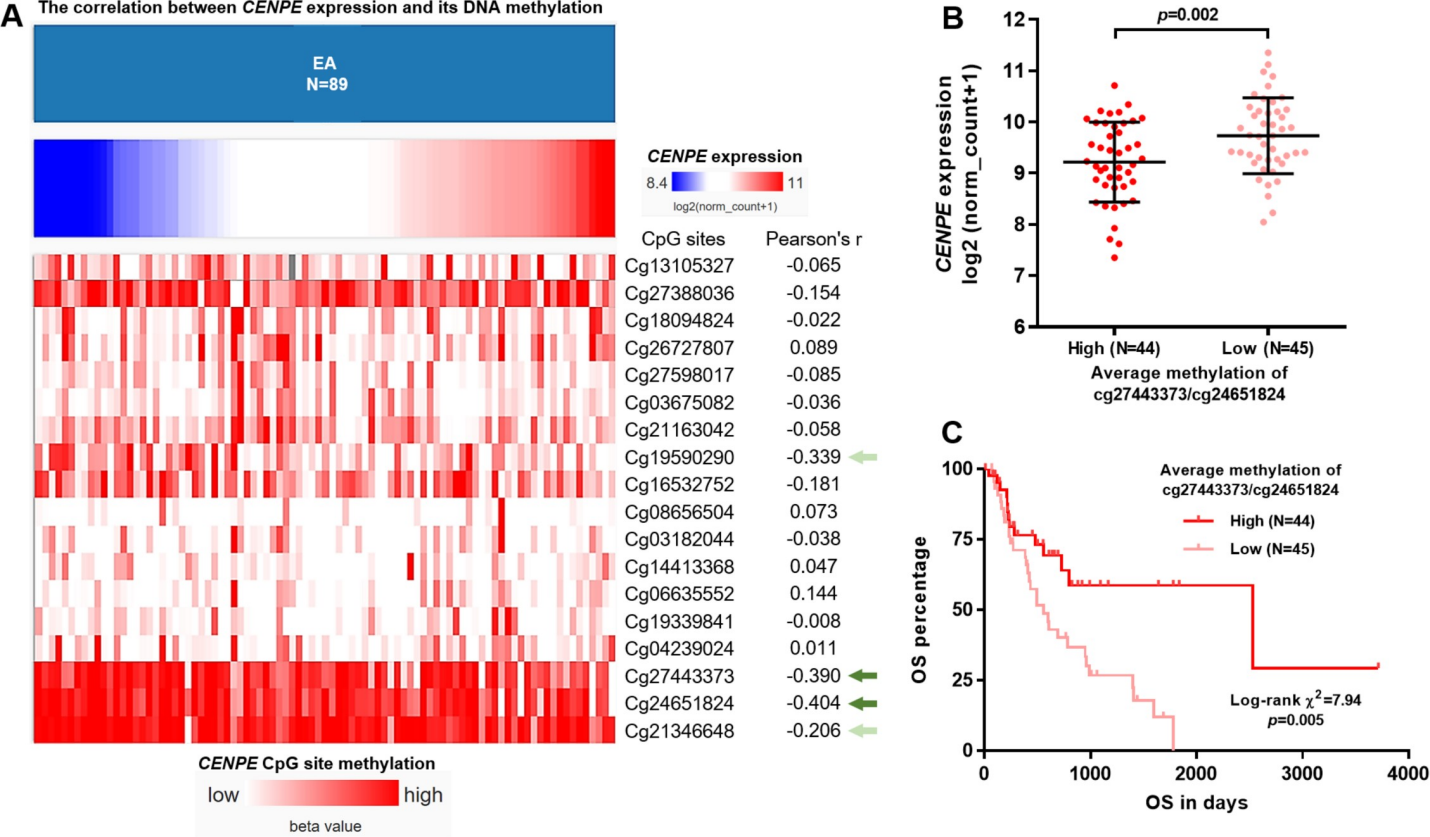
*CENPE* expression was negatively correlated with its DNA methylation in EA. **A.** A heatmap showing the correlation (represented as Pearson’s r-value) between *CENPE* expression and the methylation status of the 18 CpG sites in *CENPE* DNA in EA. **B.** Comparison of *CENPE* expression between methylation-high and methylation-low groups, according to the median average methylation of cg27443373 and cg24651824. **C.** Kaplan-Meier curves of OS of methylation-high and methylation-low groups.

By performing linear regression analysis, we identified the correlation (represented as Pearson’s r-value) between *CENPE* expression and the methylation status of the 18 CpG sites in EA samples (Fig 4A). Only the methylation level of 4 CpG sites (cg19590290, cg27443373, cg24651824 and cg21346648) were associated with *CENPE* expression (absolute Pearson’s r≥0.2) (Fig 4A, green arrows). Actually, the methylation level of 4 CpG sites were all negatively correlated with *CENPE* expression, among which cg27443373 and cg24651824 were the significantly hypomethylated sites compared with normal tissues (Fig 4A, dark green arrows). These findings suggest that these two CpG sites might play a critical role in regulating *CENPE* transcription in EA. By divided the patients into methylation-high and methylation-low groups, according to the best cutoff of the methylation of cg27443373 and cg24651824, we found that the methylation-low group had significantly higher *CENPE* expression (Fig 4B, *p* = 0.002), as well as significantly worse OS (Fig 4C, *p* = 0.005).

### *CENPE* expression in EA was not related to its CNAs

**Fig 5:**
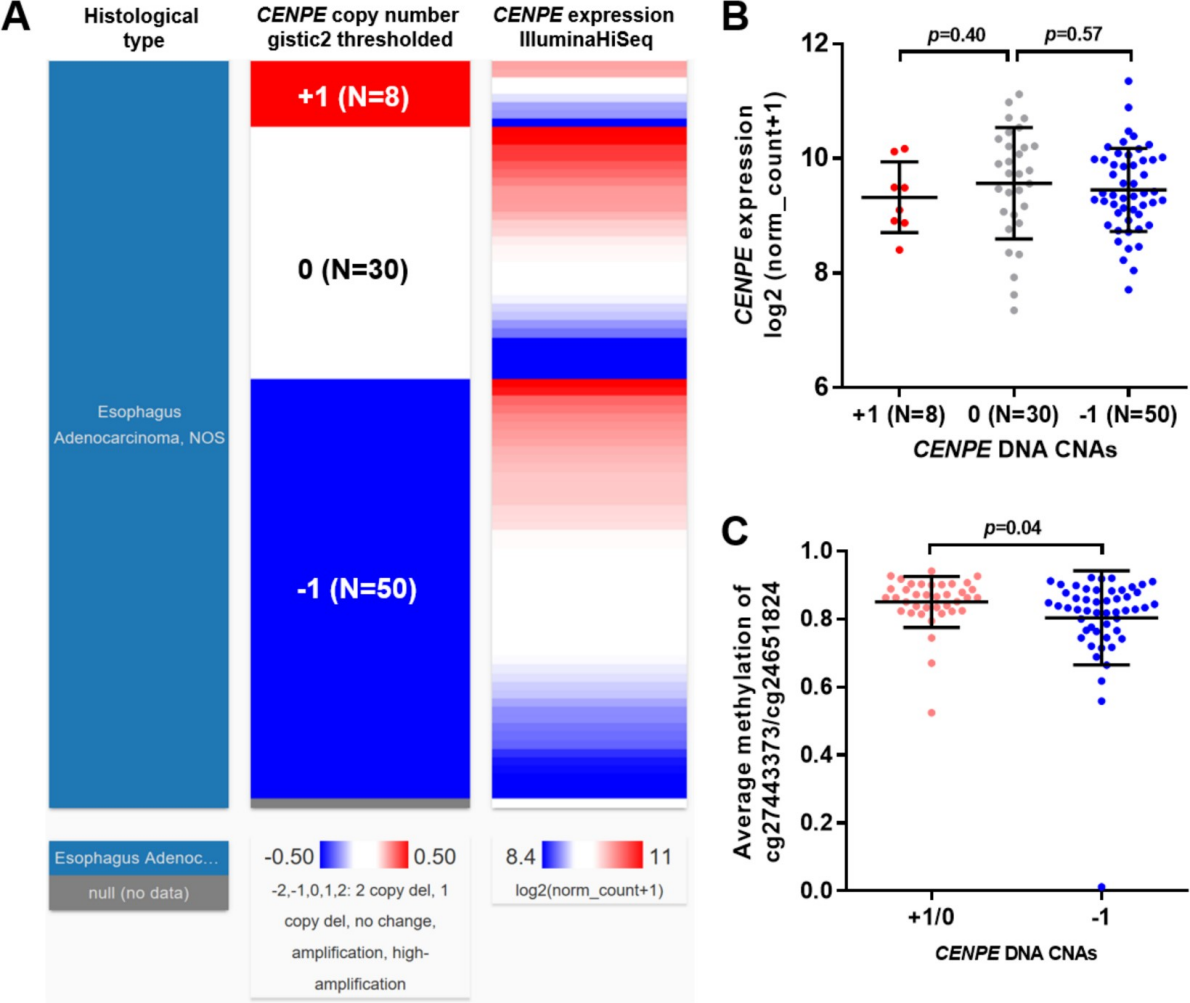
*CENPE* expression in EA was not related to its CNAs. **A-B.** A heatmap (B) and a statistical comparison summary (B) showing the correlation between *CENPE* expression and the *CENPE* copy number alterations in EA (N = 88). **C.** Comparison of the average methylation of cg27443373 and cg24651824, between amplification (+1)/copy neutral (0) cases and heterozygous loss (-1) group.

By examining *CEPNE* DNA CNAs in EA patients with copy number data available (N = 88), we observed that heterozygous loss (-1) was frequent in EA (50/88, 56.8%) (Fig 5A). However, the copy number loss was not necessarily associated with decreased *CENPE* expression compared with the copy neutral (0) cases (Fig 5B). By checking the average methylation of cg27443373 and cg24651824 between +1/0 and -1 groups, we found that the methylation of the -1 group was significantly lower than that of the +1/0 group (*p* = 0.04) (Fig 5C). These results suggest that hypomethylation might be an adaptive mechanism to compensate the influence of DNA loss on *CENPE* expression in EA.

## In silico analysis of the potential molecular actions between *CENPE* and its co-expressed genes in EA

**Fig 6:**
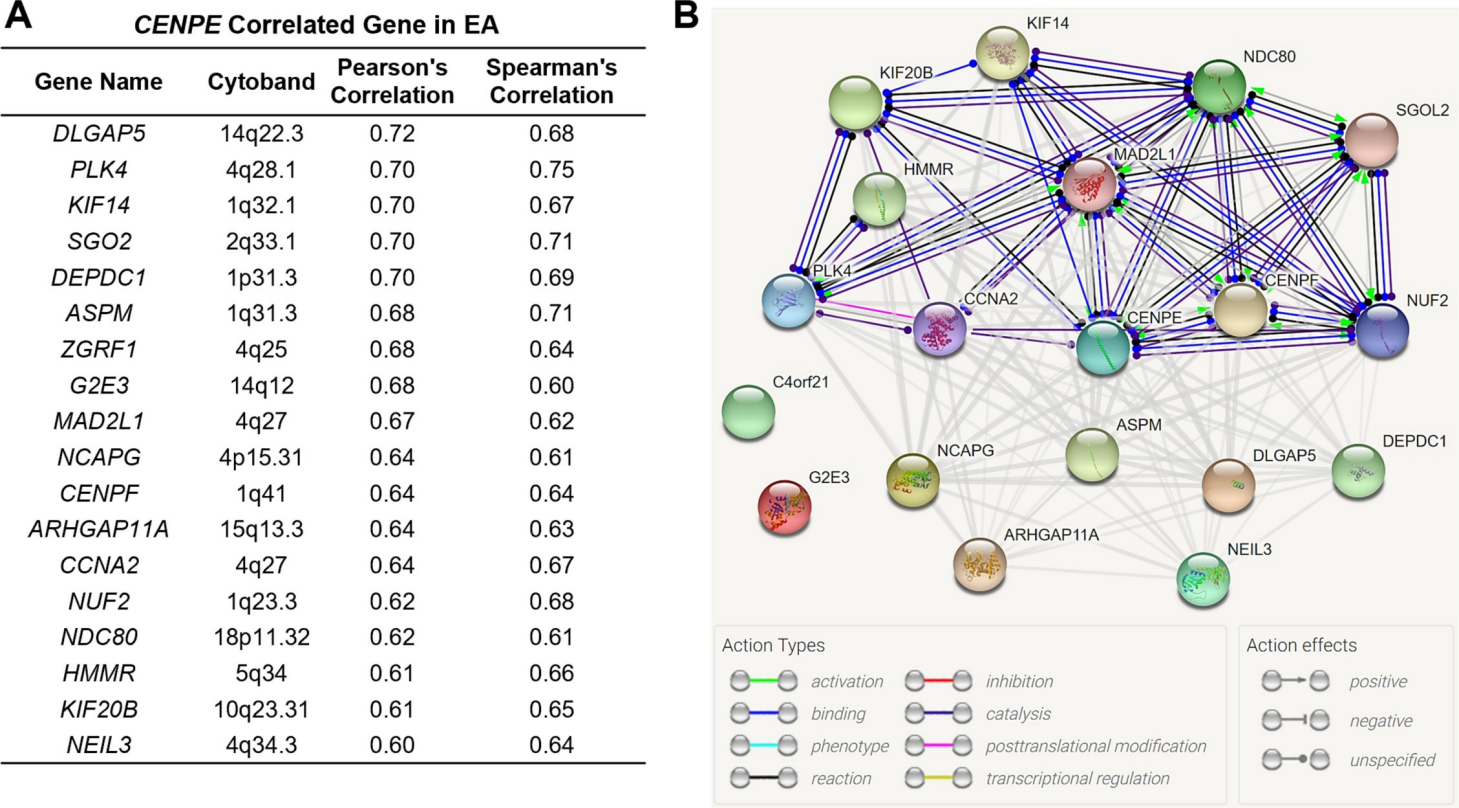
In silico analysis of the potential molecular actions between CENPE and its co-expressed genes in EA. **A.** The genes that were strongly (Pearson's r≥0.6 and Spearmen’s r≥0.6) co-expressed with *CENPE* in EA. **B.** The potential molecular associations between CENPE and its co-expressed genes.

Using the cBioPortal for cancer genomics, we identified 18 genes that were strongly (Pearson's r≥0.6 and Spearmen’s r≥0.6) co-expressed with *CENPE* in EA (Fig 6A). To explore their potential molecular associations, we performed in silico analysis using String 10.5. Results showed that CENPE might interact with KIF20B, KIF14, MAD2L1, NDC80, SGOL2, CENPF and NUF2 (Fig 6B).

## Discussion

Accurate prognostic prediction has important clinical implications since it provides fundamental information for treatment decisions and follow-up schedule. Usually, patients with favorable prognosis do not need intensive adjuvant therapy or routine follow-up. In comparison, patients with predicted unfavorable prognosis might need intensive adjuvant treatment and follow-up in a higher frequency [17]. In this study, we observed that *CENPE* was significantly upregulated in both ESCC and EA compared to that in their respective adjacent normal tissues. Kaplan-Meier curves showed that high *CENPE* expression was associated with poor OS in EA patients. The following univariate and multivariate analysis confirmed that *CENPE* expression was an independent indicator of unfavorable OS in EA patients, as a continuous variable (HR: 1.861, 95%CI: 1.235–2.806, *p* = 0.003) or as categorical variables (HR: 2.550, 95%CI: 1.294–5.025, *p* = 0.007). Based on these findings, we infer that *CENPE* expression might be a valuable prognostic biomarker in terms of OS in EA patients. However, we did not find significant prognostic value of *CENPE* expression in terms of RFS in either EA or ESCC patients.

CENPE plays a critical role in the cell-cycle progression from metaphase to anaphase [5, 18]. In breast cancer, *CENPE* upregulation was strongly and negatively correlated with disease-specific survival [13]. The progression of castration-resistant prostate cancer cells to G2-M phase is heavily dependent on CENPE [12]. Due to its critical regulative effects on cell cycle progression in cancer cells, this gene has been considered as a promising target for anticancer drugs [10, 19]. Inhibition of *CENPE* expression or selectively inhibiting CENPE motor function results in mitotic arrest due to polar chromosomes and following cell apoptosis [13]. These mechanisms help to explain why *CENPE* expression was associated with unfavorable survival in EA patients. In this study, we identified the genes that were highly co-expressed with CENPE in EA and also explored their potential molecular interactions. Among these genes, *MAD2L1* and *CENPF* are oncogenes in esophageal carcinoma [20, 21]. *CCNA2* is a cell cycle regulatory gene and its dysregulation is associated with esophageal tumorigenesis [22]. However, the potential involvement of these genes in EA and their regulatory network are largely unknown and thus need to be explored in the future.

*CENPE* expression might be induced under a stressful environment in cancer cells. For example, in triple-negative breast cancer cells, *CENPE* expression was upregulated after Docetaxel treatment [13]. In castration-resistant prostate cancer cells, the reprogramming of the binding of lysine-specific demethylase 1A (LSD1), which is a histone-modifying enzyme responsible for demethylation of histone H3 lysine 4 (H3K4), results in an increase of AR binding at the *CENPE* promoter and subsequently enhanced *CENPE* transcription [12]. These results suggest that epigenetic regulation might be a mechanism of *CENPE* dysregulation in cancer cells. In fact, epigenetic regulations, such as DNA methylation, histone acetylation, and up-regulation/down-regulation of genes by non-codling RNAs, have been characterized as important epigenetic regulations related to dysregulated tumor suppressors and other growth regulating genes during the progression of EA [23, 24]. In this study, by checking the methylation status of 18 CpG sites in *CENPE* DNA in EA, we found that three CpG sites with hypomethylated in EA, among which two sites (cg27443373 and cg24651824) showed moderately negative correlation with *CENPE* expression. Besides, the high methylation group was associated with better OS. In addition, we found that although heterozygous loss (-1) was frequent in EA (50/88, 56.8%), it was not necessarily associated with decreased *CENPE* expression. The significantly lower level of the average methylation of cg27443373 and cg24651824 in this group provides a plausible explanation of this phenomenon. Based on these findings, we infer that hypomethylation of certain CpG sites in *CENPE* DNA might be an important epigenetic mechanism of upregulated *CENPE* in EA.

This study also has some limitations. Firstly, *CENPE* protein expression in EA samples is lacking. Therefore, based on findings in this study, we can only infer the association between *CENPE* RNA expression and OS in EA patients. Secondly, we only confirmed the association between methylation and *CENPE* expression in EA. Therefore, we could not exclude the possible influence of other epigenetic regulations on *CENPE* expression. In the future, it is meaningful to explore other potential mechanisms modulating *CENPE* expression, as well as their involvement in the progression of EA.

## Conclusion

*CENPE* upregulation in EA might serve as a valuable indicator of unfavorable OS. The methylation status of cg27443373 and cg24651824 might play a critical role in modulating *CENPE* expression.

## Supporting information

S1 Fig Patient inclusion and data availability in TCGA-ESCA. (JPG)

S1 Table Univariate analysis of OS in ESCC. (DOCX)

S2 Table Univariate analysis of RFS in EA and ESCC. (DOCX)
