# Supplementary figures and images for: CENPE expression is associated with its DNA methylation status in esophageal adenocarcinoma and independently predicts unfavorable overall survival
Source: PLoS One. 2019 Feb 4;14(2):e0207341. doi: 10.1371/journal.pone.0207341 (PMC6361429; doi:10.1371/journal.pone.0207341)

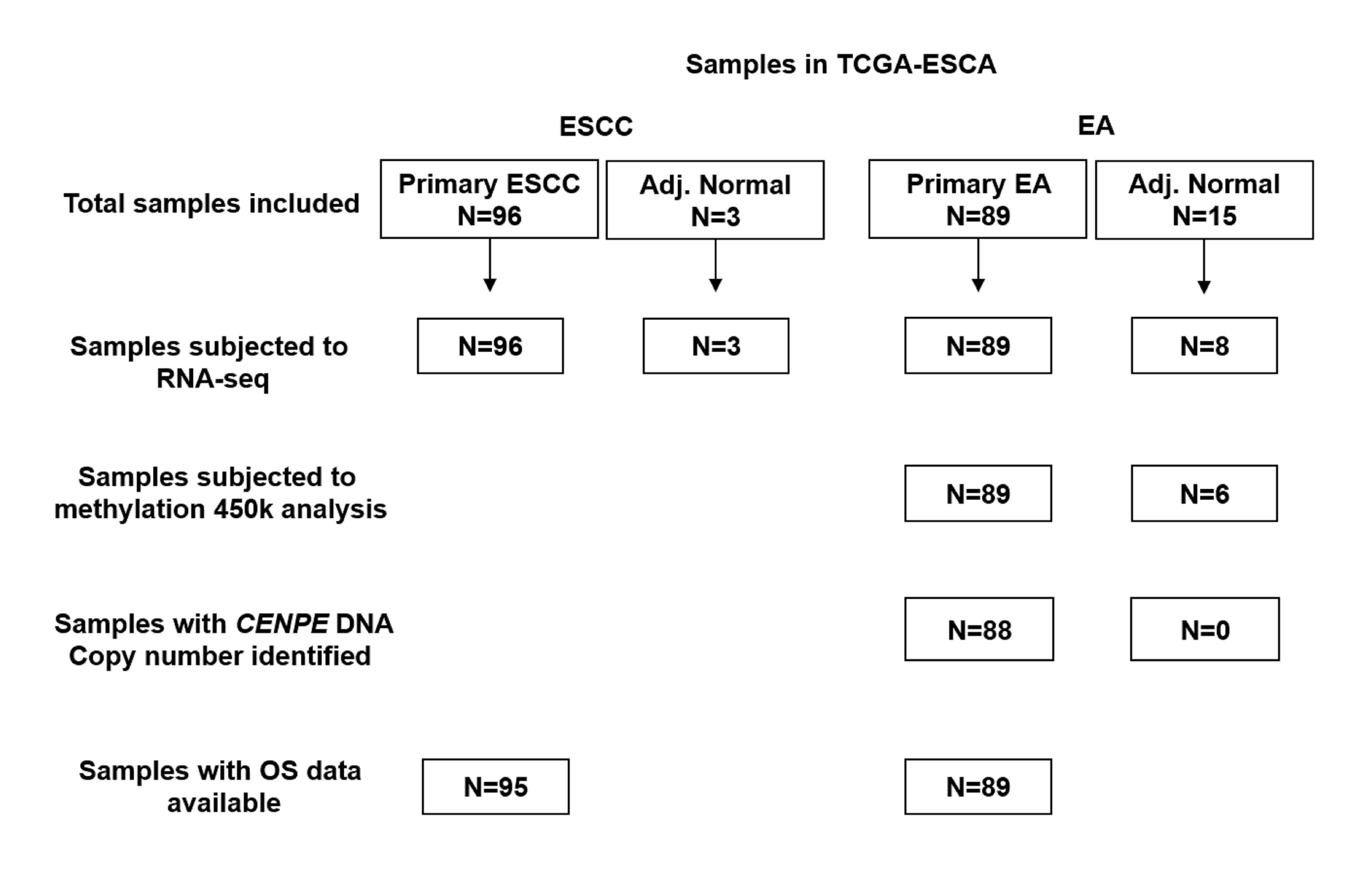

Supplement: S1 Fig — (JPG) [file pone.0207341.s001.jpg]
