# Supplementary material for: CENPE expression is associated with its DNA methylation status in esophageal adenocarcinoma and independently predicts unfavorable overall survival
Source: PLoS One. 2019 Feb 4;14(2):e0207341. doi: 10.1371/journal.pone.0207341 (PMC6361429; doi:10.1371/journal.pone.0207341)
Supplement: S1 Table — (DOCX) [file pone.0207341.s002.docx]

**S1 Table. Univariate analysis of OS in ESCC**

| **Parameters** | **Univariate analysis** | | | |
| --- | --- | --- | --- | --- |
|  | ***p*** | **HR** | **95%CI (lower/upper)** | |
| *CENPE* expression  (Continuous) | 0.120 | 0.761 | 0.539 | 1.073 |
