# Supplementary material for: CENPE expression is associated with its DNA methylation status in esophageal adenocarcinoma and independently predicts unfavorable overall survival
Source: PLoS One. 2019 Feb 4;14(2):e0207341. doi: 10.1371/journal.pone.0207341 (PMC6361429; doi:10.1371/journal.pone.0207341)
Supplement: S2 Table — (DOCX) [file pone.0207341.s003.docx]

**S2. Table Univariate analysis of RFS in EA and ESCC**

| **Parameters** | **Univariate analysis** | | | |
| --- | --- | --- | --- | --- |
|  | ***p*** | **HR** | **95%CI (lower/upper)** | |
| **EA/RFS** |  |  |  |  |
| *CENPE* expression  (Continuous) | 0.611 | 1.196 | 0.601 | 2.380 |
| **ESCC/RFS** |  |  |  |  |
| *CENPE* expression  (Continuous) | 0.765 | 0.933 | 0.590 | 1.474 |
